# Healthcare Utilization and Medical Cost of Gastrointestinal Reflux Disease in Non-tuberculous Mycobacterial Pulmonary Disease: A Population-Based Study, South Korea, 2009–2017

**DOI:** 10.3389/fmed.2022.793453

**Published:** 2022-04-18

**Authors:** Taehee Kim, Jai Hoon Yoon, Bumhee Yang, Jiin Ryu, Chang Ki Yoon, Youlim Kim, Jang Won Sohn, Hyun Lee, Hayoung Choi

**Affiliations:** ^1^Division of Pulmonary, Allergy, and Critical Care Medicine, Department of Internal Medicine, Hallym University Kangnam Sacred Heart Hospital, Hallym University College of Medicine, Seoul, South Korea; ^2^Department of Gastroenterology, Hanyang University Hospital, Seoul, South Korea; ^3^Division of Pulmonary and Critical Care Medicine, Department of Internal Medicine, Chungbuk National University Hospital, Chungbuk National University College of Medicine, Cheongju, South Korea; ^4^Biostatistical Consulting and Research Lab, Medical Research Collaborating Center, Hanyang University, Seoul, South Korea; ^5^Department of Ophthalmology, Seoul National University Hospital, Seoul, South Korea; ^6^Division of Pulmonary and Allergy, Department of Internal Medicine, Konkuk University Hospital, School of Medicine, Konkuk University, Seoul, South Korea; ^7^Division of Pulmonary Medicine and Allergy, Department of Internal Medicine, Hanyang University College of Medicine, Seoul, South Korea

**Keywords:** NTM, non-tuberculous mycobacteria, GERD (gastroesophageal reflux disease), population base study, medical cost, disease burden

## Abstract

Gastroesophageal reflux disease (GERD) is a common non-respiratory comorbidity in patients with non-tuberculous mycobacterial pulmonary disease (NTM-PD). However, little is known about the association between GERD and healthcare utilization and medical costs of NTM-PD. Thus, we evaluated this association using the Health Insurance Review and Assessment Service National Patient Sample. NTM-PD patients with GERD had significantly higher healthcare use and spent a higher total on medical costs (5,098 vs. 2,675 USD/person/year) than those without GERD (*P* <0.001 for all). Therefore, an appropriate management of GERD in NTM-PD patients can be an important factor to reduce the disease burden.

## Introduction

The prevalence and incidence of non-tuberculous mycobacterial pulmonary disease (NTM-PD) has been increasing worldwide (1). Furthermore, both the attributable mortality and financial burden associated with this disease are high (2). Thus, clinical studies that identify preventable or treatable factors to address the high disease burden of NTM-PD are urgently needed to determine strategies for reducing the burden. Gastroesophageal reflux disease (GERD) is a common non-respiratory comorbidity in NTM-PD patients, with a prevalence of 26–44% (3, 4). GERD is associated with an increased number of aspiration symptoms, higher bacterial burden, and more severe radiologic findings (3, 4). Although these results indicate that the disease burden in NTM-PD patients with GERD can be more substantial than in those without GERD, there is limited information on this issue. Thus, we aimed to evaluate whether GERD is associated with a higher disease burden, specifically healthcare use, medical costs, and in-hospital mortality, in patients with NTM-PD.

## Methods

### Data Source

We used the 2009–2017 Health Insurance Review and Assessment Service, National Patient Sample (HIRA-NPS), which includes ~1,400,000 individuals each year drawn by 3% stratified random sampling by age and sex from the entire population with claims records during the year. The dataset included patient diagnosis, treatment, procedures, surgical history, and prescription drugs. Diagnosis was coded according to the 10th edition of the International Classification of Diseases (ICD-10). Generic drugs were coded according to the Korean National Code System (5).

### Study Population

NTM-PD was defined by ICD-10 diagnosis codes of A31.0, A31.8, and A31.9; GERD was defined by ≥2 claims under the ICD-10 code K21 and proton pump inhibitors prescribed for ≥2 weeks (6). We included 6,589 patients with NTM-PD from January 2009 to December 2017 using the ICD-10 code, of which 1,407 patients had GERD. The Institutional Review Board of Chungbuk National University Hospital approved the study and waived the requirement for informed consent because the HIRA-NPS data were deidentified (application no. CBNUH 2021-03-020).

### Outcomes

Respiratory-related healthcare use was defined as healthcare use under the ICD-10 codes of respiratory diseases (J00–J99). We compared healthcare use (outpatient department [OPD] visits, emergency room [ER] visits, or hospitalizations), medical costs, and in-hospital mortality in NTM-PD patients with GERD to those in patients without GERD. The medical cost consists of expenses related to diagnostic tests, procedures, and treatments covered by the National Health Insurance (7).

### Covariables

Comorbidities were also defined using ICD-10 codes. Pulmonary comorbidities were defined as chronic obstructive pulmonary disease (COPD; J42–J44, except J43.0 [unilateral emphysema]), asthma (J45–J46), bronchiectasis (J47, excluding E84 [cystic fibrosis] or Q33.4, Q89.3 [congenital bronchiectasis]), pulmonary tuberculosis (TB; A15–19), and lung cancer (C33–C34). Extrapulmonary comorbidities were defined as cerebrovascular disease (G45–G46, I60–I69, and H34.0), hypertension (I10–I15), angina or myocardial infarction (MI; I20, I21, I22, and I25.2), congestive heart failure (I43, I50, I09.9, I11.0, I25.5, I13.0, I13.2, I42.0, I42.5–I42.9, and P29.0), inflammatory bowel disease (K50–K51), diabetes mellitus (E10–E14), chronic kidney disease (N18), and connective tissue disease (M05, M06, M315, M32, M33, M34, M351, M353, and M360) (5, 8). The Charlson comorbidity index (CCI) was calculated using a modified version consisting of 17 comorbidities (9).

### Statistical Analysis

We calculated the age-adjusted prevalence of GERD among patients with NTM-PD by dividing the number of events by 1,000 person-years. All categorized variables were compared using Pearson's chi-squared test. To compare the medical use of NTM-PD patients with and without GERD, logistic regression analysis was used to determine the odds ratio (OR) for healthcare use (ER visits or hospitalizations) and in-hospital mortality in NTM-PD patients with GERD relative to those without GERD. A multivariable model was adjusted for age, sex, type of insurance (health insurance, medical aid, and veteran status), bronchiectasis, and CCI (0–1 and ≥2). All statistical analyses were performed with SAS 9.4 (SAS Institute, Cary, NC, USA). Statistical significance was set at *P* < 0.05.

## Results

### Baseline Characteristics

The baseline characteristics of the patients are summarized in Table 1. The prevalence of NTM-PD in patients with GERD was significantly higher in older age groups (≥60 years [60.4 vs. 42.8%], *P* < 0.001). However, there was no difference in the sex ratio (~62% of the cases were female). The proportion of patients who received medical aid was higher in the NTM-PD with GERD group than in those without GERD (7.3 vs. 3.8%, *P* < 0.001). Analysis of comorbidities indicated that the rates of asthma (40.9 vs. 25.7%), chronic obstructive pulmonary disease (37.5 vs. 23.0%), bronchiectasis (28.9 vs. 24.1%), hypertension (44.1 vs. 27.6%), diabetes mellitus (34.0 vs. 19.0%), cerebrovascular disease (15.7 vs. 7.8%), connective tissue disease (12.3 vs. 4.5%), and CCI ≥ 2 (78.2 vs. 49.0%) were significantly higher in NTM-PD patients with GERD than in those without GERD (*P* < 0.001 for all).

**Table 1:** Characteristics of study population.

	**Total (*N* = 6,589)**	**NTM-PD with GERD (*n* = 1,407)**	**NTM-PD without GERD (*n* = 5,182)**	***P*-value**
**Age, years**				<0.001
20–29	499 (7.6)	25 (1.8)	474 (9.2)	
30–39	877 (13.3)	96 (6.8)	781 (15.1)	
40–49	789 (12.0)	125 (8.9)	664 (12.8)	
50–59	1,360 (20.5)	311 (22.1)	1,049 (20.1)	
60–69	1,407 (21.4)	403 (28.6)	1,004 (19.4)	
≥70	1,657 (25.2)	447 (31.8)	1,210 (23.4)	
**Sex**				0.614
Male	2,458 (37.3)	533 (37.9)	1,925 (37.2)	
Female	4,131 (62.7)	874 (62.1)	3,257 (62.8)	
**Type of insurance**				<0.001
Health insurance	6,283 (95.3)	1,302 (92.5)	4,981 (96.2)	
Medical aid	301 (4.6)	102 (7.3)	199 (3.8)	
Veteran status	5 (0.1)	3 (0.2)	2 (0.0)	
**Comorbidities**				
COPD	1,720 (26.1)	528 (37.5)	1,192 (23.0)	<0.001
Asthma	1,909 (29.0)	575 (40.9)	1,334 (25.7)	<0.001
Bronchiectasis	1,653 (25.1)	406 (28.9)	1,247 (24.1)	<0.001
Cerebrovascular disease	626 (9.5)	221 (15.7)	405 (7.8)	<0.001
Hypertension	2,051 (31.1)	620 (44.1)	1,431 (27.6)	<0.001
Diabetes mellitus	1,464 (22.2)	478 (34.0)	986 (19.0)	<0.001
Connective tissue disease	405 (6.2)	173 (12.3)	232 (4.5)	<0.001
**Charlson comorbidity index**				<0.001
0–1	2,952 (44.8)	307 (21.8)	2,645 (51.0)	
≥2	3,637 (55.2)	1,100 (78.2)	2,537 (49.0)	

*Data are presented as numbers (percentages).*

*NTM-PD, non-tuberculous mycobacterial pulmonary disease; GERD, gastroesophageal reflux disease; COPD, chronic obstructive pulmonary disease*.

### Prevalence of GERD in Patients With NTM-PD

Of the 6,589 patients with NTM-PD, 1,407 had GERD. During the study period, the prevalence of GERD in patients with NTM-PD was 21.4%, which was higher than that in patients aged ≥20 years (7.4%) in the HIRA-NPS database. The prevalence of GERD in patients with NTM-PD increased with age.

### Healthcare Use and Medical Costs According to the Presence or Absence of GERD

**Figure 1:**
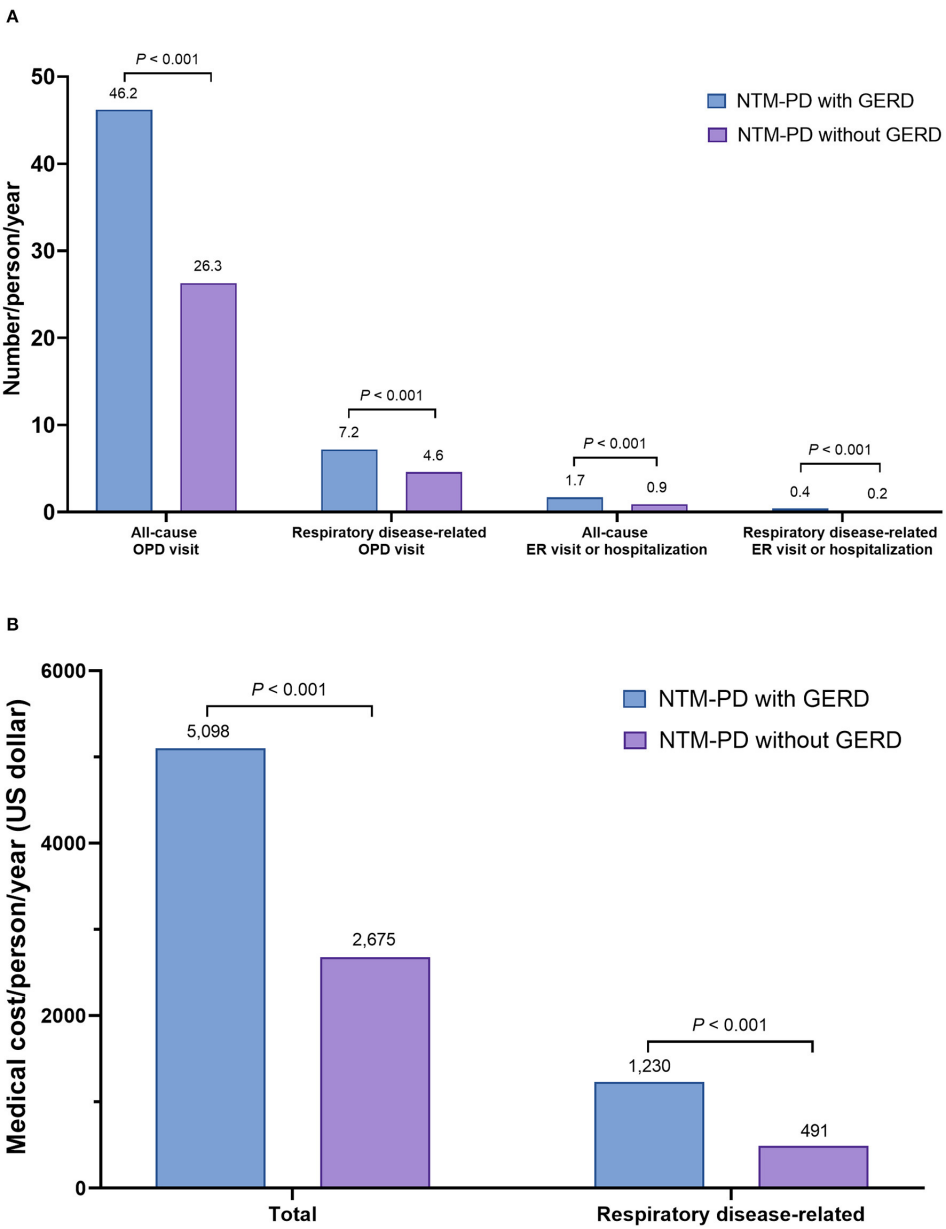
Disease burden of GERD in patients with NTM-PD: **(A)** healthcare use of patients with NTM-PD according to presence or absence of GERD (number of events/person/year), and **(B)** medical costs of patients with NTM-PD according to presence or absence of GERD (medical cost/person/year, unit US dollar). NTM-PD, non-tuberculous mycobacterial pulmonary disease; GERD, gastroesophageal reflux disease; OPD, outpatient department; ER, emergency room.

NTM-PD patients with GERD had significantly higher healthcare use, including all-cause and respiratory disease-specific OPD visits and ER visits or hospitalizations, than those without GERD (*P* < 0.001 for all). Furthermore, NTM-PD patients with GERD spent a higher total on medical costs (5,098 vs. 2,675 USD/person/year), including respiratory disease-related costs (1,230 vs. 491 USD/person/year) (*P* < 0.001 for both) (Figure 1).

### Association Between GERD and Increased Healthcare Use

**Figure 2:**
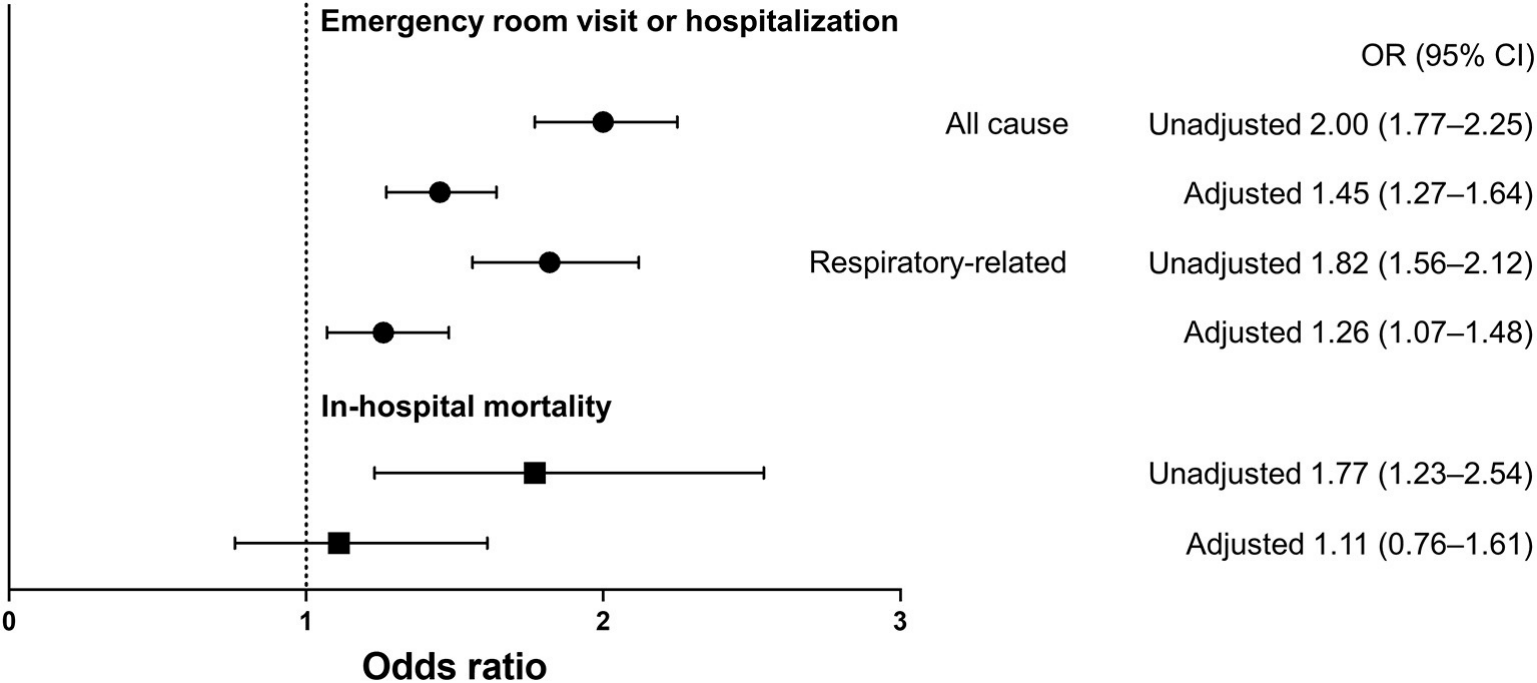
Odds ratio and 95% confidence interval for emergency room visits, hospitalization, and in-hospital mortality in NTM-PD patients with GERD relative to those without GERD. The multivariable model was adjusted for age, sex, type of insurance (health insurance, medical aid, and veteran status), bronchiectasis, and Charlson comorbidity index (0–1 and 2). NTM-PD, non-tuberculous mycobacterial pulmonary disease; GERD, gastroesophageal reflux disease; ER, emergency room; OR, odds ratio; CI, confidence interval.

GERD was independently associated with more ER visits or hospitalizations in patients with NTM-PD: all-cause (adjusted OR, 1.45; 95% confidence interval [CI], 1.27–1.64) and respiratory disease-related (adjusted OR, 1.26; 95% CI, 1.07–1.48). However, GERD was not significantly associated with in-hospital mortality (adjusted OR 1.11, 95% CI, 0.76–1.61) (Figure 2).

## Discussion

To our knowledge, this study is the first to evaluate the impact of GERD on the estimated disease burden based on healthcare use, medical costs, and in-hospital mortality in patients with NTM-PD. NTM-PD patients with GERD had significantly higher healthcare use and medical costs than those without GERD; however, there were no significant intergroup differences in in-hospital mortality.

GERD is prevalent in patients with NTM-PD, affecting up to 44% of patients (3, 4). In agreement with previous reports, 21% of patients with NTM-PD had GERD, which was significantly higher than the prevalence of GERD (7%) in the overall population aged ≥20 years during the study period. GERD has been recognized not only as an underlying disease for the occurrence of NTM-PD (10) but also as an important comorbidity that can influence the symptoms and severity of the disease (3, 10). In this regard, this study revealed NTM-PD patients with GERD are hampered by greater healthcare use and higher medical costs, which denote a significant disease burden, compared to those without GERD.

Gastro-esophageal refluxate can aggravate respiratory disease by either stimulating the sensitized esophageal-bronchial neuronal pathway or by microaspiration, which involves aspiration into the airway (11). Conversely, it has been suggested that progression of chronic lung disease can lead to deterioration of GERD (12). Consequently, it can be postulated that the interaction between the two diseases may further deteriorate the health of patients with NTM-PD and GERD. Considering the potential bidirectional process between reflux disease and chronic respiratory disease (13), appropriate treatment of GERD may lead to decreased disease burden and improvement in overall treatment outcomes in patients with NTM-PD. However, since acid reduction cannot reduce refluxate other than acid, there is a possibility that non-acid refluxate can still cause lung damage. Thus, it should be acknowledged that it is challenging to reduce reflux itself.

Our study has several strengths that support our hypothesis and was based on a large, nationally representative database. However, there are some limitations to this study. First, we used a cross-sectional study design; thus, long-term mortality was not fully evaluated based on the presence or absence of GERD. Second, there is a possibility of misclassification because we used the ICD-10 codes to define NTM-PD and GERD. Therefore, a large prospective cohort study is needed to clarify the role of GERD in NTM-PD.

In conclusion, GERD significantly increased healthcare use and medical costs in patients with NTM-PD. Thus, appropriate diagnostic and management plans to reduce the GERD-associated disease burden would bring significant improvements to patients with NTM-PD.

## Data Availability Statement

Publicly available datasets were analyzed in this study. This data can be found here: https://opendata.hira.or.kr/op/opc/selectPatDataAplInfoView.do.

## Author Contributions

HL and HC were responsible for the conception and design of the study. TK, JHY, BY, JR, CKY, YK, JWS, HL, and HC undertook the analysis and interpretation of the data. TK, JHY, BY, HL, and HC drafted the manuscript. All authors made a critical revision of the manuscript, and read and approved the final manuscript.

## Funding

This work was supported by a National Research Foundation of Korea (NRF) grant funded by the Ministry of Science, Information, and Communications Technologies (Nos. 2020R1F1A1070468 and 2021M3E5D1A0101517621 to HL) and the Korean Ministry of Education (No. 2021R1I1A3052416 to HC). The funders had no role in the design of the study, the collection and analysis of the data, or the preparation of the manuscript.

## Conflict of Interest

The authors declare that the research was conducted in the absence of any commercial or financial relationships that could be construed as a potential conflict of interest.

## Publisher's Note

All claims expressed in this article are solely those of the authors and do not necessarily represent those of their affiliated organizations, or those of the publisher, the editors and the reviewers. Any product that may be evaluated in this article, or claim that may be made by its manufacturer, is not guaranteed or endorsed by the publisher.
